# Effect of thermal annealing on an emissive layer containing a blend of a small molecule and polymer as host for application in OLEDs

**DOI:** 10.1039/d3ra06271f

**Published:** 2023-11-16

**Authors:** Bushra Basharatali Meer, Dhruv Sharma, Swapnil Tak, Gauri Govardhan Bisen, Mahendra D. Shirsat, Kalpathy Ganapathy Girija, Sanjay Sanatan Ghosh

**Affiliations:** a Optoelectronics Laboratory, Department of Physics, Kavayitri Bahinabai Chaudhari North Maharashtra University Jalgaon-425001 Maharashtra India ssghosh@nmu.ac.in; b Chemistry Division, Bhabha Atomic Research Centre Mumbai 400085 India; c RUSA Centre for Advanced Sensor Technology, Department of Physics, Babasaheb Ambedkar Marathwada University Aurangabad-431004 Maharashtra India

## Abstract

In order to improve the performance of OLEDs, a host–guest mixture was used as an emissive layer. To have better host properties, a mixture of different materials with suitable properties can also be used as a host. In this study, we used a mixture of a polymer and a small molecule as the host and studied the effect of thermal annealing on the emissive layer properties by using Ir(ppy)_3_ as the emitter. UV-visible absorption, steady-state and time-resolved photoluminescence, scanning electron microscopy, atomic force microscopy, and optical microscopic studies were performed to study the film properties. Devices were fabricated and their current–voltage and luminance–voltage characteristics were studied. Charge-carrier mobility in the devices was studied by dark CELIV and transient electroluminescence methods. We show that, below the glass transition temperature of the polymer, the small molecules formed aggregates due to thermal annealing, which was beneficial for the device performance in the lower-temperature range, mainly due to the improved electron mobility. However, this aggregate formation was detrimental in the higher-temperature range, as it led to inefficient energy transfer due to the increased pure phase formation. At temperatures above the glass transition temperature of the polymer, the small molecules were seen to be distributed more uniformly into the polymer matrix. However, as a result of the degradation of the polymer property due to degradation of the primary chain of the phenyl ring of the polymer, this uniform distribution was not of any use and the device performance deteriorated.

## Abstract 2

Morphology changes upon thermal annealing the emissive layer and aggregation behavior of the two hosts is shown.

## Graphical abstract

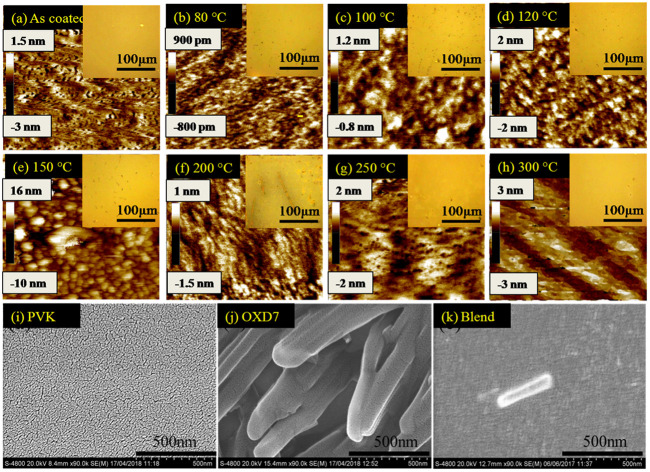

## 1. Introduction

Organic light emitting diodes (OLEDs) are gaining much attention because of their various advantages, such as simple and low cost processing potential, light weight, durability, high power efficiency, full-color display, eco-friendliness, superior color quality, wide viewing angle, mercury-free manufacturing, and fascinating flexibility.^1^ An OLED is composed of at least one organic layer that is put between two electrodes. Electrons and holes are injected from either sides of the electrode. After applying a voltage, both carriers will drift toward the other side in the applied electrical field. When both electrons and holes meet at the bulk or the interface, they recombine to form excitons, which will decay to produce light emission.^2^ In 1987, Tang *et al.* and VanSlyke reported OLEDs based on small organic molecules by thermal vacuum deposition.^3^ Device efficiency and functionality can be controlled by engineering the carrier-transport properties of organic semiconductors. The transport properties can be manipulated by optimizing the morphological structure, doping, or by a magnetic or electric field and temperature.^4^ Generally the emissive layer consists of a blend of a host and guest. There are reports of a blend of materials having superior electron and mobility separately being used as the host. The post-deposition processing method offers an opportunity to decouple film formation from structural development, providing greater control over the molecular ordering in organic semiconductor thin films. Thermal annealing treatment has played an important role in easily controlling device performance.^5^ The general interpretation of such an annealing effect on a device is that it causes an enhancement in the interfacial bonding between the substrate and the organic layers, which promotes the surface planarity of the organic films. It has been reported that the evaporation of solvent may create empty spaces for the inter-chains to move while thermal annealing at temperatures higher than the glass transition temperature (*T*_g_) would result in a relaxation of the chains.^6^ In addition, the annealing of the polymers at a temperature higher than the *T*_g_ but lower than the boiling temperature of the solvent would result in a better packing.^7^ A *vice versa* condition would otherwise enhance the relaxation of the polymer chain. Also, when using a polymer host and small molecule iridium complex as an emitter, annealing at 200 °C led to a chemical gradient of Ir(ppy)_3_ molecules, while at the same time their original needle-type morphology, formed in the as-processed state, was sustained. Understanding the mechanism responsible for the temperature-dependent performances of the emitting layer is essential for OLEDs fabricated by solution-processable methods. Burns *et al.* found a strong co-relation between the thermal annealing temperature and the performance of OLEDs by using a super yellow emissive layer.^8^ Lee *et al.* reviewed the use of mixed hosts for obtaining high efficiency and long lifetime OLEDs.^9^ They explained the importance of using two hosts. S. A. Jenekhe's group studied various commercially available electron-transport materials by using a mixed host.^10^ In spite of all these studies, however, we were not able to find any reports where a dedicated thermal annealing study of host blends was performed by using a blend of a polymer and small molecule.

In this work, we used a mixture of a polymer and small molecule as the host and studied the effect of thermal annealing from room temperature up to 300 °C on the emissive layer properties. Considering that a large difference in the molecular weights can give strikingly different aggregation properties, the polymer PVK was used as a p-type host, while the small molecule OXD-7 was used as an n-type host and Ir(ppy)_3_ as the emitter. With the use of separate hosts for electron and hole transport, the hole and electron transport took place independently in the p- and n-type hosts. The carrier injection and transport in the emitting layer could be relatively easily compared to that in a single host.^9^ Both the host molecules were chosen so that their emission overlapped with the absorption of the emitter.^11^ This creates a favorable situation for Forster-type energy transfer between the host and the guest. The emissive layer properties were studied by UV-visible absorption, steady-state and time-resolved photoluminescence, scanning electron microscopy (SEM), atomic force microscopy (AFM), and optical microscopy (OM). Devices were fabricated and their current–voltage (*JV*) and luminance–voltage (*LV*) characteristics were studied. Charge-carrier mobility in the devices was studied by dark charge extraction by linearly increasing voltage (CELIV) and transient electroluminescence methods. We show that due to the thermal annealing, the small molecules had a tendency to form aggregates. This was beneficial for the device performance up to an annealing temperature of 120 °C, but was detrimental at higher-temperature ranges due to inefficient energy transfer. At temperatures above the glass transition temperature of the polymer, the small molecules were observed to be distributed more uniformly into the polymer matrix. At temperatures above 250 °C, the polymer property diminished, which is detrimental to device performance. We also found that the electron mobility, which was nearly two orders of magnitude less than the hole mobility, determined the device performance.

## 2. Experimental details

### 2.1. Device fabrication and characterization techniques

PVK : OXD-7 (1 : 1) as the host and Ir(ppy)_3_ as the dopant for the ternary blend-based OLED were fabricated through a solution-processing method. An ITO-coated glass substrate was cleaned by sonication in distilled water, acetone, and isopropanol for 15 min and by vacuum drying for 10 min at 150 °C. Next a hole-injection layer (HIL) with a thickness of 40 nm was deposited by spin-coating. The hole-injection layer (HIL) composed of poly(ethylenedioxythiophene):polystyrenesulfonate (PEDOT:PSS) and 2-propanol in a volume ratio of 1 : 2.5 was spin-coated at 1500 rpm for 30 s, and then transferred to the glove box after annealing at 150 °C for 15 min. The emissive layer of the ternary blend (PVK:OXD-7:Ir(ppy)_3_) in chlorobenzene solvent with 2% dopant concentration and a thickness of 70 nm was spin-coated on the HIL. For the annealing study, the samples were thermally annealed by transferring to a preheated hot plate at 80 °C, 100 °C, 120 °C, 150 °C, 200 °C, 250 °C, and 300 °C for 10 min. After annealing, BPhen dissolved in a mixture of formic acid and water with a 3 : 1 ratio was spin-coated to obtain a 30 nm layer and backed at 50 °C as the EIL. A 100 nm thick aluminum cathode layer was progressively deposited under vacuum conditions of 2 × 10^−6^ torr for the fabrication of the OLEDs. Pre-patterned indium-tin oxide (ITO)-coated glass substrate with a sheet resistance of <15 Ω per square and transmittance of >90% were purchased from Xinyan Technology, Taiwan. (PEDOT:PSS) Al 4083 was purchased from H. C. Starck, Clevios P. The electron-transport layer (BPhen), hole-transporting host PVK with an average molecular weight of ∼1 100 000 g mol^−1^, and the phosphorescent guest Ir(ppy)_3_ were purchased from Sigma-Aldrich. OXD7 of >99% purity was purchased from LumTec., Taiwan. If not stated all other chemicals and solvents were purchased from Sigma-Aldrich. The UV-visible absorption spectra of all the samples were measured on an absorption spectrometer (Shimadzu 1601). The steady-state photoluminescence (PL) spectra were recorded using a Horiba spectrophotometer in the reflectance mode. The time-resolved photoluminescence (TRPL) spectra were recorded using an FLSP920 system (Edinburgh Instruments). The samples were excited with a hydrogen-filled nanosecond flash lamp operating at 6.8 kV and 40 kHz pulse frequency. The film morphology was studied by field effect scanning electron microscopy (FE-SEM; Hitachi S4822). AFM topography images and roughness values were obtained in tapping mode by using an instrument from Park Systems Korea (Model-XE7). Optical microscopy (OM) images were recorded by using an Olympus BX60 instrument. Current–voltage characteristics were recorded with a Keithley source-measuring unit (Model 2611A). Luminance was measured using a luminance meter LS-160 from Konica Minolta. Electroluminescence (EL) spectra were recorded by a spectrometer (CXR-25; StellarNet). The thickness of the different layers was determined using a Dektak 150 profilometer. The dark CELIV and transient electroluminescence studies were performed using PAIOS 4.O (Fluxim). All the measurements were carried out at room temperature.

## 3. Result and discussions

### 3.1. Optical properties of the films

**Fig. 1:**
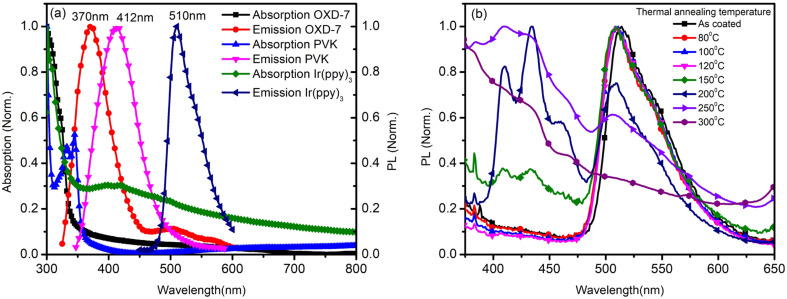
(a) Normalized absorption and photoluminescence spectra of the films for OXD7, PVK, and Ir(ppy)_3_. (b) Normalized photoluminescence spectra of the blend thin film after annealing at different temperatures.

The annealing temperature studied in the present case was a much larger range from room temperature to 300 °C. Due to this large temperature range, the temperature was divided into two ranges. First, the low-temperature range (room temperature to 120 °C) and second, the high-temperature range (120 °C to 300 °C). Fig. 1(a) shows the absorption and photoluminescence spectra of the films of the two hosts PVK, OXD7, and the guest Ir(ppy)_3_. The n-type host OXD7 film had an emission peak at 370 nm, while that of the p-type host PVK was obtained at 412 nm. For the guest Ir(ppy)_3_, the absorption onset was at 510 nm and extended to the ultraviolet region. Thus the emission of both host molecules overlapped with the absorption of the guest, which is very much favorable for Forster-type energy transfer between the host and the guest. Fig. 1(b) shows the normalized PL spectra of the blend emissive layer PVK-OXD7:Ir(ppy)_3_ with 2% dopant concentration. The as-coated film displayed a peak at 514 nm. The emission of the hosts at 412 nm and 370 nm were thus effectively suppressed by the guest. The dominant peak at 514 nm was slightly red-shifted to 510 nm by annealing the emissive layer at 80 °C, and this peak remained at this point even after annealing at higher temperature up to 200 °C. This shift signified the improvement in Ir(ppy)_3_ ordering in the film due to reorganization at elevated temperature.^12^ Upon increasing the annealing temperature to 250 °C, the peak further shifted to 506 nm. Apart from this shift in guest emission, the host emission started to appear in the 150 °C annealed film. The intensity with respect to the guest emission increased with increasing temperature. At 300 °C, negligible guest emission was seen, which indicated there was nearly no energy transfer from the host to the guest. Through this study, it was concluded that in the lower-temperature range up to 120 °C, reorganization of the components in the blend film took place without any loss related to Forster-type energy transfer.^13^ At 150 °C and above, enhanced reorganization takes place, but energy-transfer losses started to appear, which increased with increasing the temperature.

**Fig. 2:**
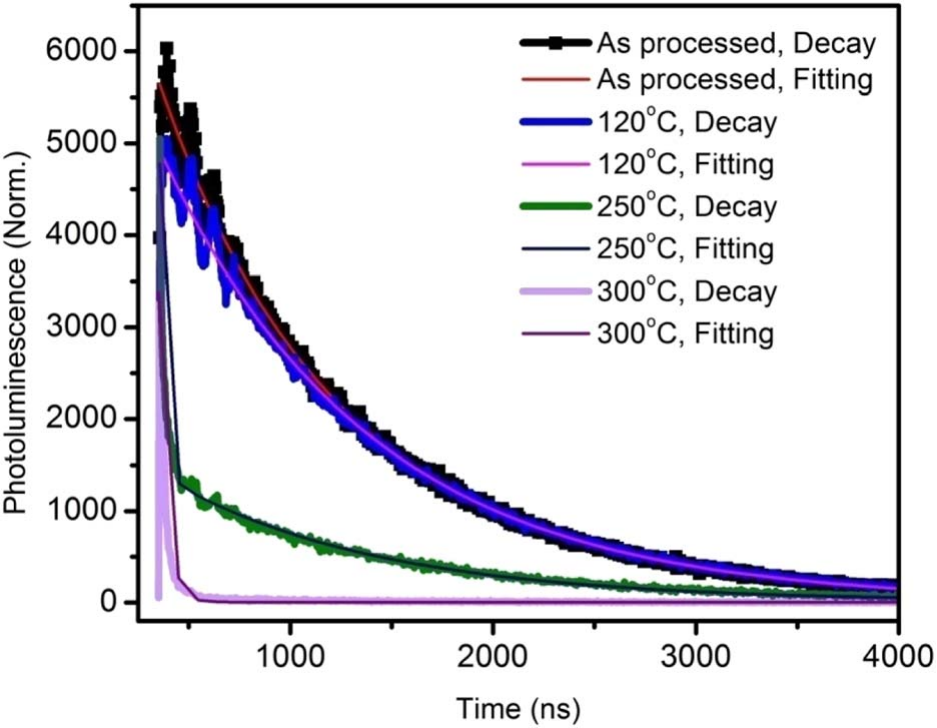
Time-resolved photoluminescence decay plots and curve fittings of the active layer annealed at various temperatures.

To gain a further understanding of the films, time-resolved photoluminescence spectra were recorded and fitted using double-exponential functions, as shown in Fig. 2.

The corresponding lifetimes and the percentage decay of each lifetime are given in Table 1. It can be seen that the average lifetime of the film annealed at 120 °C was longer than that of the as-processed film. By increasing the annealing temperature, the lifetime decreased drastically. This indicated the poor emission property of the films due to the thermal annealing at higher temperatures.^14^ At 300 °C annealing temperature, the lifetime was a very low 11 ns.

**Table 1:** Decay times *τ*_1_, *τ*_2_ and relative amplitudes *f*_1_ and *f*_2_ of the films annealed at different temperatures

Annealing temperature (°C)	*τ*_1_ (ns)	*f*_1_	*τ*_2_ (ns)	*f*_2_	*τ*_av_(ns)
As-processed	567	0.3	1150	0.7	993
120	1036	0.5	1036	0.5	1036
250	—	—	21	1	21
300	—	—	11	1	11

### 3.2. Morphology of the films

**Fig. 3:**
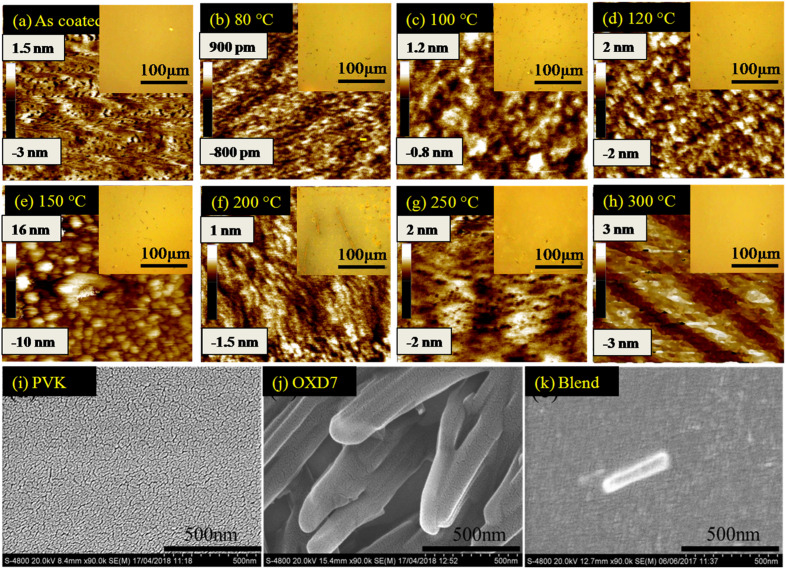
(a–h) AFM topography images (tapping mode) of the blend films annealed at various temperatures; inset shows the corresponding optical microscope images. (i–k) SEM images of pure PVK, OXD7 and the blend films. Scale of AFM images is 500 nm × 500 nm.

To investigate the reason for this emission behavior and the corresponding changes in the morphology at the micrometer and nanometer scale, OM, AFM, and SEM images were recorded, and are shown in Fig. 3. The OM images showed that at the tens of micrometer scale, the film was featureless in the as-coated film. Aggregates were seen when annealing at 80 °C, the size of which increased when annealing up to 150 °C. The 200 °C-annealed film showed a large growth in the structures and rod-like structures were seen sized more than 100 μm. Thus it was concluded that enhancing the temperature resulted in the growth of nucleation sites, the size of which increased with increasing the temperature. Correlating this with Fig. 1(b), it seems that distance between the excited host and guest molecules was too large due to the aggregation and formation of pure phases by increasing the annealing temperature to 150 °C, which led to a loss in energy transfer to the guest molecules.^15^ Since Ir(ppy)_3_ is a small molecule with a molecular weight of 654.8, it could be expected to form such nucleation sites and hence aggregation. However, the n-type host OXD7 was also a small molecule with a molecular weight of 478.6 and it was also expected to form such aggregates seen in the optical microscopy images.^16^ To confirm this, SEM images were recorded for PVK (Fig. 3(i)) and OXD7 (Fig. 3(j)) films without Ir(ppy)_3_. It was found that the PVK films area was overall featureless with no large features, while the OXD7 film showed rod-like structures similar to those in the blend films (Fig. 3(k)). A closer look at the emission spectra of the films annealed at 150 °C and 200 °C showed the dominant PL emission of PVK only and no significant increase in emission of OXD7 at 390 nm. We attributed this to the decrease in OXD7 from the bulk of the polymer PVK matrix due to formation of larger OXD7 aggregates. With the increase in temperature above 120 °C, the PL intensity decreased and shoulder peaks appeared due to the increase in new defect levels, which caused a change in the peak position. Furthermore, non-radiative recombination among the electrons and holes increased, which reduced in intensity with the increase in temperature.

The AFM images showed the nano-scale morphology of the films (Fig. 3). For the film annealed at 80 °C, shown in Fig. 3(b), the height difference and also the rms roughness were lower than for the as-coated film in Fig. 3(a). This showed the smoothening of the blend films upon annealing at lower temperatures. Upon further increasing the temperature, both the height difference and rms roughness increased slightly when annealing beyond the low-temperature range up to 150 °C (Fig. 3(c)–(e)). The OM images in the inset also showed the formation of bigger and more aggregates in the films. At 200 °C (as shown in Fig. 3(f)), however, rod-like structures could be seen in the OM images. These structures disappeared at higher temperatures. The glass transition temperature (*T*_g_) of the polymer PVK was between 200–250 °C.^17^ The thermal annealing of such blends above the *T*_g_ can make polymer materials exhibit a fluid-like behavior, and hence movement of the polymer chains is activated. The disappearance of the larger structures in the films annealed at 250 °C, as shown in Fig. 3(g), and 300 °C, as shown in Fig. 3(h), was due to homogeneous mixing of the small molecules in the soft PVK matrix. The reduction in roughness of the films at these temperatures was also attributed to this factor. The steady-state emission, as shown in Fig. 1(b), showed an enhanced emission intensity of OXD7 compared to PVK at these temperatures. This is an indication of the good intermixing of the small molecules in the PVK matrix. At 200 °C, however, the height difference between the valleys and peaks, as shown in the AFM images, was very low. This was in contrast with the OM images, where large rod-like features could be observed. This may be due to the fact that the temperature was slightly below the glass transition temperature of PVK. This reduction in roughness may be due to the fact that the bulk of the blend was devoid of small molecules due to the formation of large aggregates in certain positions. Therefore, the reduced roughness was attributed to the reduction of small molecules from the PVK matrix due to the formation of tens of micrometer-sized structures. This led to the formation of purer phases in the blend films and hence lower roughness.

### 3.3. Transient electroluminescence, CELIV, and device studies

#### 3.3.1. Transient electroluminescence

**Fig. 4:**
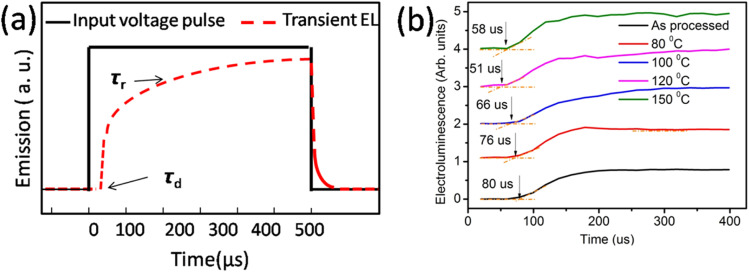
(a) Input voltage pulse and expected electroluminescence and (b) actual electroluminescence rise in the device.

In transient EL measurements, the time-dependent EL response is monitored upon exciting the OLEDs with a rectangular voltage pulse. The input voltage pulse used here was 12 volt, with a pulse length of 500 μs, settling time of 200 μs, and follow-up time of 100 μs, as shown in Fig. 4(a).

The finite delay time (*τ*_d_), which is between the application of a voltage pulse and the onset of EL signal, can be determined by the arrival of the slower charge carrier of the injected carriers at the emission zone.^18^ Therefore *τ*_d_ is the electron-transporting time in the emissive layer, which is nearly same for all devices. Electron mobility is calculated by using the formula,
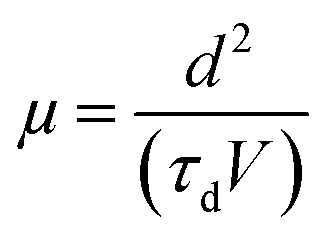
where *d* is the thickness of the emitting layer, *V* is the driving voltage, and *τ*_d_ is the delay time. The calculated electron mobilities at various annealing temperatures are summarized in Table 2.

**Table 2:** Delay time *τ*_d_, saturation time*τ*_r_, and mobility *μ* for devices with the active layer annealed at: (1) as-processed, (2) 80 °C, (3) 100 °C, (4) 120 °C, (5) 150 °C

Annealing temperature (°C)	*τ*_d_ [μs]	*τ*_r_ [μs]	Mobility (*μ*) [cm^2^ V^−1^ s^−1^]
As-processed device	80	206	(5.0 ± 0.2) × 10^−8^
80 °C	59	204	(5.2 ± 0.2) × 10^−8^
100 °C	60	202	(6.1 ± 0.2) × 10^−8^
120 °C	60	298	(7.9 ± 0.3) × 10^−8^
150 °C	59	178	(6.9 ± 0.2) × 10^−8^

Table 2 shows the tabulated mobility values calculated according to Fig. 4(b), showing that the electron mobility increased in the lower-temperature range and decreased in the higher-temperature range.

#### 3.3.2. CELIV studies

**Fig. 5:**
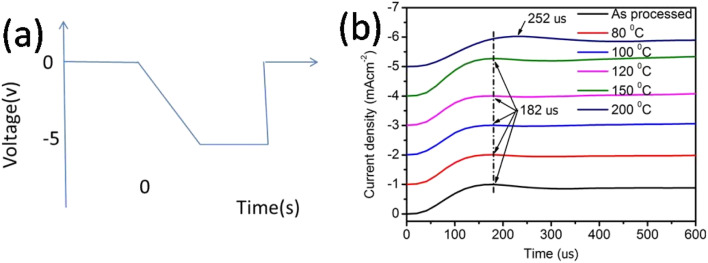
(a) Input pulse and the (b) experimental CELIV curve.

Generally, the hole mobility is roughly 2–3 orders of magnitude higher than the electron mobility in OLEDs.^19^ It is therefore expected that the carrier mobility determined by the CELIV technique would be hole mobility. In this experiment, mobile charge carriers that are present in the device were extracted by a reverse voltage ramp (triangular pulse). For the input offset voltage of 0 V, and voltage of 5 V, an injection time of 22.3 ms was used. A representative pulse is shown in Fig. 5(a).

In all CELIV techniques, the characteristic current peak area, namely, the time integral of the current overshoot, can be analyzed to estimate the amount of accumulated charge in the device. The peak time *t*_max_ is related to the charge-carrier mobility *μ* following the analytically derived formula:^20^
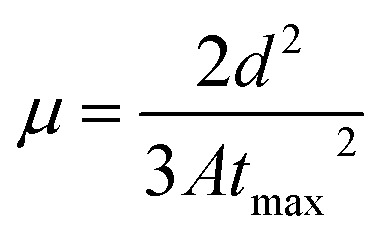
where *d* is the thickness of the device and *A* is the employed voltage ramp. Here, we used *d* = 70 nm, *A* = 2.81 V ms^−1^.

Table 3 shows that the hole mobility (calculated by using the data in Fig. 5(b)). It shows that the hole mobility did not change with respect to the annealing temperature up to 150 °C. At 200 °C, the mobility was found to decrease. At higher annealing temperatures, we were not able to get reliable CELIV data.

**Table 3:** Lifetime measurement for the active layer of the devices annealed at different temperatures

Annealing temperature (°C)	Mobility (μ) in cm^2^ V^−1^ s^−1^
As-processed device	(5.26 ± 0.14) × 10^−7^
80	(5.26 ± 0.19) × 10^−7^
100	(5.26 ± 0.23) × 10^−7^
120	(5.26 ± 0.29) × 10^−7^
150	(5.26 ± 0.35) × 10^−7^
200	(2.74 ± 0.38) × 10^−7^

#### 3.3.3. Device studies and discussion

**Fig. 6:**
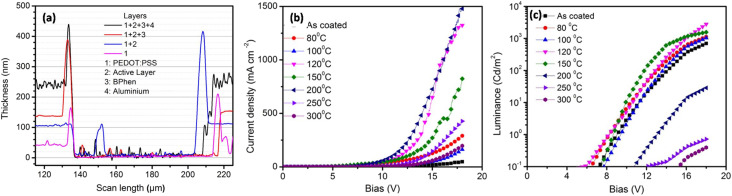
(a) Thickness of different layers of device coated on glass substrates, (b) current–voltage characteristics, (c) luminance–voltage characteristics of the fabricated devices.

While recording the *JV* and *LV* characteristics, voltage was applied such that holes were injected from the ITO electrode into the PEDOT:PSS buffer layer and electrons were injected from the Al electrode into the BPhen buffer layer. Crossing these two, carriers entered the emissive layer PVK:OXD-7:Ir(ppy)_3_, where electrons and holes recombined to emit light. Fig. 6(a) shows the thicknesses of the different layers coated on glass substrates and measured by using a profilometer, while Fig. 6(b) shows the current–voltage characteristics of the fabricated devices and Fig. 6(c) shows the luminance–voltage characteristics of the fabricated devices. Thicknesses were measured by making a scratch in the films using a sharp edge. The emissive layer was annealed at different temperatures. It can be seen that with increasing the annealing temperature, the turn-on voltage of the devices was reduced and the maximum luminance increased in general in the lower-temperature range. At 120 °C-annealing temperature, the lowest turn-on voltage and maximum luminance were achieved. In the higher-temperature range, however, both the turn-on voltage and maximum luminance increased. The better performance of the device after annealing at 120 °C was attributed to a balance among the better morphology, carrier mobility, and efficient energy transfer from the host to the guest.^21^ Under this condition, the films were rougher than the as-coated film and that annealed at lower temperatures. The charge-transfer property remained nearly unchanged in this temperature range. Therefore the improved performance in the lower-temperature range with the increase in annealing temperature was attributed to the better electron mobility with increasing the annealing temperature.^22,23^ The hole mobility was similar in all the films. The increased roughness was due to the formation of pure phases due to aggregation of the small molecules. However, this small aggregation, on the other hand, improved the electron mobility, leading to an overall improvement in device performance. In the higher-temperature range, the device performance was degraded. In this temperature range, a number of other parameters come in to play to decide the overall device performance. The steady-state and TRPL studies suggested a reduction in change transfer. Also, the transient electroluminescence studies showed reduced electron mobility and the AFM study showed an increases film roughness at 150 °C. The steady-state PL emission showed the signature of the host emission at this temperature. Thus inefficient energy transfer took place at this temperature. We therefore attribute the reduction in device performance to the reduced electron mobility as well as inefficient charge transfer.^24^ At 200 °C and higher temperature, the device performance was also poor. At this temperature, we were not able to record the electroluminescence due to the low emission. At 250 °C and also at 300 °C, the device performance was even poorer. We attribute this to the degradation in PVK molecules. The reduced emission of PVK (around 410 nm as compared to that of OXD7) in Fig. 1(b) at these temperatures indicated the poor emission from PVK.

## 4. Conclusion

We used a mixture of a polymer and small molecule as the host and studied the effect of thermal annealing on the emissive layer properties. Due to thermal annealing, the small molecule formed aggregates below the glass transition temperature of the polymer. This is beneficial for the device performance in the low-temperature range, but is detrimental at 150 °C and higher temperatures, mainly due to inefficient energy transfer. At temperatures above the glass transition temperature of the polymer, the small molecules were seen to be distributed more uniformly in the polymer matrix. However at temperatures above 250 °C, the polymer property was diminished due to degradation of the primary chain of the phenyl ring of the polymer, which is detrimental to device performance.

## Conflicts of interest

The authors declare no conflicts of interest.

## Supplementary Material
